# What Are the Barriers and Enablers to the Implementation of Pharmacogenetic Testing in Mental Health Care Settings?

**DOI:** 10.3389/fgene.2021.740216

**Published:** 2021-09-22

**Authors:** Adam Jameson, Beth Fylan, Greg C. Bristow, Gurdeep S. Sagoo, Caroline Dalton, Alastair Cardno, Jaspreet Sohal, Samantha L. McLean

**Affiliations:** ^1^Bradford District Care NHS Foundation Trust, Bradford, United Kingdom; ^2^School of Pharmacy and Medical Sciences, University of Bradford, Bradford, United Kingdom; ^3^Wolfson Centre for Applied Health Research, Bradford, United Kingdom; ^4^Bradford Institute of Health Research, NIHR Yorkshire and Humber Patient Safety Translational Research Centre, Bradford, United Kingdom; ^5^Academic Unit of Health Economics, Leeds Institute of Health Sciences, University of Leeds, Leeds, United Kingdom; ^6^National Institute for Health Research Leeds in vitro Diagnostics Co-operative, Leeds Teaching Hospitals NHS Trust, Leeds, United Kingdom; ^7^Biomolecular Sciences Research Centre, Sheffield Hallam University, Sheffield, United Kingdom; ^8^Leeds Institute of Health Sciences, Faculty of Medicine and Health, University of Leeds, Leeds, United Kingdom

**Keywords:** pharmacogenomics, pharmacogenetics, pharmacogenetic testing, psychiatry, mental health

## Abstract

In psychiatry, the selection of antipsychotics and antidepressants is generally led by a trial-and-error approach. The prescribing of these medications is complicated by sub-optimal efficacy and high rates of adverse drug reactions (ADRs). These both contribute to poor levels of adherence. Pharmacogenetics (PGx) considers how genetic variation can influence an individual’s response to a drug. Pharmacogenetic testing is a tool that could aid clinicians when selecting psychotropic medications, as part of a more personalized approach to prescribing. This may improve the use of and adherence to these medications. Yet to date, the implementation of PGx in mental health environments in the United Kingdom has been slow. This review aims to identify the current barriers and enablers to the implementation of PGx in psychiatry and determine how this can be applied to the uptake of PGx by NHS mental health providers. A systematic searching strategy was developed, and searches were carried out on the PsychInfo, EmBase, and PubMed databases, yielding 11 appropriate papers. Common barriers to the implementation of PGx included cost, concerns over incorporation into current workflow and a lack of knowledge about PGx; whilst frequent enablers included optimism that PGx could lead to precision medicine, reduce ADRs and become a more routine part of psychiatric clinical care. The uptake of PGx in psychiatric care settings in the NHS should consider and overcome these barriers, while looking to capitalize on the enablers identified in this review.

## Introduction

Prior to the COVID-19 pandemic the estimated social and economic cost of mental health illness in England was £119 billion a year (O’Shea and Bell, 2020). Mental Health (MH) illnesses are a leading cause of health burden worldwide (Vos et al., 2015). Medication can treat those with mental illnesses; antidepressants and antipsychotics are commonly prescribed. However, in some patients the benefit of such pharmacological interventions is hindered by a lack of therapeutic response and significant adverse drug reactions (ADRs) (Sinyor et al., 2010; Huhn et al., 2019). Approximately 30% of people diagnosed with schizophrenia are treatment resistant and similarly up to 50% of people prescribed an antidepressant do not respond initially (Meltzer, 1997; Fava, 2003; Lally et al., 2016). An estimated 40% of people taking antidepressants will experience ADRs (Bull et al., 2002). Antipsychotics can cause a variety of adverse effects (Huhn et al., 2019). Limited efficacy and ADRs, combined with the relapsing-remitting nature of many mental illnesses, contributes to poor adherence to these drug classes (Semahegn et al., 2020). Furthermore, non-adherence to medication is a predictor of poorer health outcomes in MH patient populations, with increased risk of relapse (Leucht et al., 2012; Kato et al., 2021).

Pharmacogenomics is the study of how genetic variation can influence response to medication (Roden, 2006). These variations are often single nucleotide polymorphisms (SNPs), where a substitution occurs at a single base in the genome resulting in a different genetic sequence (Butler, 2012). Previous studies suggest that over 90% of the population carry at least one change that may alter drug response (Van Driest et al., 2014; Bush et al., 2016; Ji et al., 2016; Mostafa et al., 2019). PGx has the potential to make a significant impact in the field of psychiatry. The NHS has seen prescribing of psychotropic medication increase in recent years (Wilson, 2020). Approximately 10% of the United Kingdom adult population are prescribed an antidepressant (Taylor et al., 2019). During the COVID-19 pandemic, antidepressant prescribing in the NHS increased by four million items, leading to a £140 million increase in spending compared with the previous year (Rabeea et al., 2021). Antipsychotics are a mainstay of treatment in schizophrenia. The selection of these medications is largely driven by a trial-and-error approach. The implementation of PGx in the field of psychiatry has the potential to improve this, by moving current practice toward a more personalized approach of prescribing, considering patient genetic profiles. There is hope that this could increase the efficacy and reduce adverse drug reactions (ADRs) associated with psychotropic medications (Manchia et al., 2020).

**FIGURE 1:**
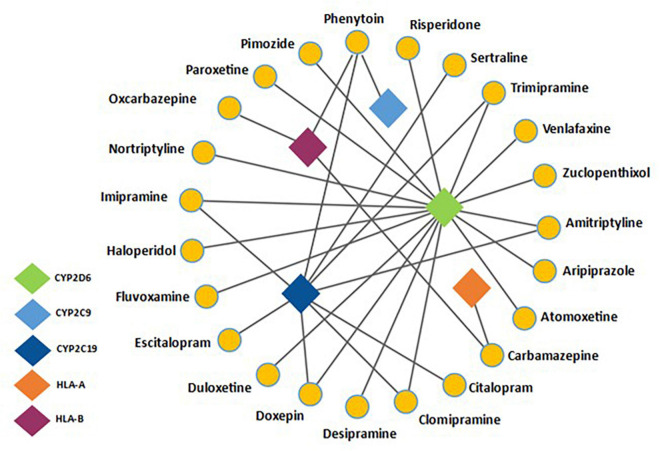
Adapted from Bousman C. et al. (2019) and information presented on https://www.pharmgkb.org/- a network analysis detailing common psychotropic drugs with their associated pharmacogenes. CYP2D6 and CYP2C19 are common metabolic pathways for a range of antidepressants and antipsychotics. Also noted is the drug-gene interaction between HLA-B and Phenytoin/Carbamazepine, a well-documented pharmacogenetic interaction.

Pharmacogenetics can influence drug response in two ways, either by altering the pharmacokinetics or pharmacodynamics of a drug (Roden, 2006). Focus so far in psychiatric PGx has been on pharmacokinetics. The cytochrome P450 family has been well studied (Bousman et al., 2021) and are an important group of metabolic enzymes, with humans possessing over 30 isoenzymes, of which six are responsible for metabolizing over 90% of drugs (Stavropoulou et al., 2018). In psychiatric PGx, the genes coding for the CYP2D6 and CYP2C19 isoenzymes have been studied most extensively (van Westrhenen et al., 2020). An estimated 36% and 62% of people worldwide have enzyme variants that affect the function of these two enzymes, respectively (Koopmans et al., 2021). Most psychotropics are metabolized by these enzymes; as such genetic variations of these enzymes can impact upon the pharmacokinetics of psychotropic medications (Hiemke et al., 2018). This can result in poor therapeutic response if the active compound is cleared too quickly, or an increased risk of ADRs if there is a reduced capacity to clear the drug from the plasma (Fleeman et al., 2011). So far, the evidence base for actionability, based on pharmacodynamic pharmacogenes is limited. Common psychotropic drug-gene interactions can be seen in Figure 1.

Knowledge of which drugs are implicated by drug-gene interactions (DGIs), caused by genetic variations in pharmacogenes is increasing (Peters et al., 2014; England, 2016; Maggo et al., 2019; Pratt et al., 2019, 2021). PharmGKB^1^ and CPIC^2^ databases provide information on DGIs, such as the evidence base supporting DGIs and suggested actions where applicable. The recommendations are graded by strength of evidence. Several pharmacogenetic tests are now available, however only a limited number of tests are relevant to psychiatry. Bousman C. et al. (2019) suggested a minimum genetic testing panel for use in psychiatry should include 16 allelic variants across five genes (CYP2D6, CYP2C9, CYP2C19, HLA-A, and HLA-B) (Bousman C. et al., 2019). Recommendations for CYP2C19, CYP2C9, and CYP2D6 genotyping have also been put forward (Pratt et al., 2018, 2019, 2021).

Despite examples of localized PGx use in MH clinical practice in some countries, to our knowledge the uptake of PGx in MH settings in the United Kingdom has been limited to date (Maggo et al., 2019). In the United Kingdom, the NHS now has a strategy to implement personalized medicine as part of a wider policy effort to increase the uptake of genomic medicine. The NHS genomic medicine service has been set up to help deliver this and aims for PGx to be a routine part of NHS care by 2025 (England, 2016). Before this can happen, the way in which PGx is integrated into the NHS and its position in clinical care pathways needs to be investigated. Implementation science can play a key role in ensuring that the uptake of PGx has a positive impact on clinical practice (Peters et al., 2014).

This mini review, therefore, aims to look at how PGx can potentially be implemented in psychiatry, by identifying enablers that help facilitate the uptake of PGx in mental health and determine factors acting as barriers to the integration of PGx in mental health care settings.

## Methods

This mini review used a systematic search strategy to identify suitable studies for inclusion. It adhered to the Preferred Reporting Items for Systematic Review (PRISMA) principles (Page et al., 2021) (see Supplementary Appendix 1). A Population, Intervention, Comparator, Outcome, Settings (PICOS) tool was used to identify the relevant keywords and Medical Subject Headings (MeSH) words (Miller and Forrest, 2001).

Inclusion criteria were developed prior to undertaking the searches, to select the most appropriate studies for the review. Both qualitative and quantitative studies collecting primary data from healthcare professionals and patients exploring viewpoints and perspectives on implementing PGx in psychiatry were included. Conference abstracts, expert opinion, anecdotal pieces, and articles not written in English were excluded, as were controlled trials and cohort studies, as the purpose of the review was not to evaluate the clinical utility of using PGx in psychiatry, but rather to identify potential enablers and barriers to its implementation.

Between April 14th and July 7th 2021, PubMed, EmBase, and PsychInfo databases were searched. The following terms were used in the searches: “pharmacogenomics,” “pharmacogenetics,” “pharmacogenetic testing,” “psychiatry,” “mental health,” and “mental illness.” One researcher (AJ) screened the titles and abstracts of the search results. Following this, the methods of included studies were evaluated to determine the type of data collected. Once appropriate papers were identified, full texts were screened and included papers were critically appraised. In total 11 papers were identified as appropriate to be included (see Supplementary Appendix 2).

A Microsoft Office Excel spreadsheet, adapted from the Critical Appraisal Skills Programme (CASP) checklist for qualitative studies checklist was developed to facilitate data extraction and critical appraisal (Long et al., 2020) by one researcher (AJ). For each of the included studies, the following data was extracted and stored in the spreadsheet: title, author, publication year, aims, sample size and characteristics, data collection method, data analysis method, study findings, strengths, limitations, barriers, and enablers.

Barriers and enablers identified from both qualitative and quantitative primary data, were synthesized into key themes and sub-themes using thematic analysis, by one researcher (AJ) following discussions between AJ, BF, and SM. These themes are presented as sub-headings in the Section “Results.” Key themes are presented as individual sub-headings, while sub-themes that emerged from a limited number of included studies or that did not link to a key theme, were grouped together in a miscellaneous group presented in Sections “Miscellaneous: Trust, Discrimination and Replacing Clinical Judgment” for barriers and “Miscellaneous: Improving Patient Experience With Medications” for enablers. This review summarizes these findings and, in the discussion, offers suggestions about how these identified barriers and enablers can be considered in the NHS as PGx is integrated into psychiatric clinical care in the United Kingdom in the future.

## Results

By evaluating the papers included in this review, a range of barriers and enablers to implementing PGx in psychiatry were identified. These were categorized into four main barrier themes and four main enabler themes, listed in order of how strongly these themes emerged from the studies. Sub-themes were also identified, that although relevant, emerged less commonly from the studies selected. There was a total of three sub-themes identified as barriers and two sub-themes identified as enablers. A summary of the barriers and enablers extracted from the included studies can be found in Table 1.

**TABLE 1:** Summary of barriers and enablers extracted from each study.

**Barriers:**	
Cost or funding	Dunbar et al. (2012), Shishko et al. (2015), Chan et al. (2017), Liko et al. (2020), Laplace et al. (2021)
Lack of knowledge and current evidence base about PGx	Shishko et al. (2015), Chan et al. (2017), Vest et al. (2020), Laplace et al. (2021)
Incorporation into current workflow	Dunbar et al. (2012), Shishko et al. (2015), Goodspeed et al. (2019), Vest et al. (2020)
Misinterpretation of results	Liko et al. (2020), Vest et al. (2020)
Fear of discrimination based on PGx results	McCarthy et al. (2020), Laplace et al. (2021)
Concerns PGx could replace clinical judgment	Dunbar et al. (2012), Laplace et al. (2021)
Trust in PGx results vendor	Goodspeed et al. (2019)
Lack of clear guidelines about PGx in psychiatry	Chan et al. (2017)
Risk of psychological distress for patients	Laplace et al. (2021)

**Enablers:**	

Perception PGx will lead to precision prescribing	Dunbar et al. (2012), Thompson et al. (2015), Shishko et al. (2015), Chan et al. (2017), Liko et al. (2020), Vest et al. (2020)
Interest in the use of PGx	Shishko et al. (2015), Chan et al. (2017), Liko et al. (2020), McCarthy et al. (2020), Vest et al. (2020), Kastrinos et al. (2021)
Aid clinician-patient relationship	Goodspeed et al. (2019), Vest et al. (2020), Dunbar et al. (2012)
Belief PGx will become a routine part of practice	Shishko et al. (2015), Thompson et al. (2015), Walden et al. (2015), Laplace et al. (2021)
Belief PGx can help reduce ADRs	Dunbar et al. (2012), Shishko et al. (2015), Laplace et al. (2021)
Low risk of psychological distress by receiving PGx results	McCarthy et al. (2020), Kastrinos et al. (2021)
Hope that PGx can guide prescribing decisions	McCarthy et al. (2020)
Belief that PGx could be useful for those with previously unsuccessful treatment	Laplace et al. (2021)

Of the included studies, seven were conducted in the United States (Shishko et al., 2015; Thompson et al., 2015; Goodspeed et al., 2019; Liko et al., 2020; McCarthy et al., 2020; Vest et al., 2020; Kastrinos et al., 2021), with the remaining studies being conducted in Canada (Walden et al., 2015), France (Laplace et al., 2021), Singapore (Chan et al., 2017), and New Zealand (Dunbar et al., 2012). Three of the studies gathered patient perspectives about PGx (Liko et al., 2020; McCarthy et al., 2020; Kastrinos et al., 2021), while the remaining studies gathered HCPs viewpoints on PGx (Dunbar et al., 2012; Shishko et al., 2015; Thompson et al., 2015; Walden et al., 2015; Chan et al., 2017; Goodspeed et al., 2019; McCarthy et al., 2020; Vest et al., 2020; Laplace et al., 2021). Data collection methods varied with some studies opting for focus groups (Goodspeed et al., 2019; Vest et al., 2020), while one conducted semi-structured interviews (Liko et al., 2020) and other studies collected data using a survey (Shishko et al., 2015; Thompson et al., 2015; Walden et al., 2015; Kastrinos et al., 2021) or questionnaire (Dunbar et al., 2012; Chan et al., 2017; McCarthy et al., 2020; Laplace et al., 2021). The study setting varied also, with the majority of studies being conducted in secondary care settings (Dunbar et al., 2012; Shishko et al., 2015; Thompson et al., 2015; Walden et al., 2015; Chan et al., 2017; Goodspeed et al., 2019; Liko et al., 2020; McCarthy et al., 2020), while one was carried out in both primary and secondary care settings (Vest et al., 2020), and two studies were unspecified (Kastrinos et al., 2021; Laplace et al., 2021). A summary of the sample, data collection methods, barriers and enablers for each study can be found in Supplementary Appendix 2.

### Barriers

#### Cost and Funding of Pharmacogenetics

Several studies found that both patients and healthcare professionals (HCPs) had concerns over the cost of PGx. In a United States study by Liko et al. (2020), cost influenced patients’ decision-making: one cited that if the costs were lower or covered by insurance, they would be more inclined to have the test done. It was also highlighted that paying for the test may be perceived as more beneficial if the patient has tried and failed several pharmacotherapies. Likewise, clinicians in Singapore surveyed by Chan et al. (2017) discovered over 94% of respondents were concerned about cost, making it the biggest perceived risk of PGx testing within the survey. A similar survey among United States psychiatric pharmacists by Shishko et al. (2015), showed a lack of funding or expense to be the main reason for not offering PGx testing, with only a third of respondents agreeing that benefits outweigh the costs from a clinical perspective. Cost was also raised as an issue by clinicians who were interviewed after ordering a PGx test for use in a United States psychiatric clinic in a study by Dunbar et al. (2012). Likewise, in the study of French psychiatrists by Laplace et al. (2021), 86% of respondents raised cost as a barrier. In Dunbar et al. (2012); Chan et al. (2017), Laplace et al. (2021), the context of which cost was perceived as a barrier was not explored.

#### Lack of Knowledge, Evidence Base, and Clinical Guidelines About Pharmacogenetics

Concerns were raised by mental health (MH) providers regarding clinicians’ lacking the knowledge about PGx required to implement PGx into their practice. Vest et al. (2020) conducted focus groups with MH prescribers, both psychiatrists and family doctors in the United States. They cited a lack of knowledge about the evidence supporting PGx use in psychiatry and a limited current evidence base. United States psychiatric pharmacists surveyed by Shishko et al. (2015) mirrored these points, with nearly half of respondents perceiving themselves as completely or somewhat unaware about PGx. In the same way, psychiatrists and psychiatric pharmacists surveyed by Chan et al. (2017) expressed how they lack knowledge about PGx in psychiatry, beyond the role of HLA testing in Carbamazepine prescribing. A lack of clear guidelines was also identified as a barrier to clinical implementation. Laplace et al. (2021) echoed these findings, with 61% of respondents claiming they lacked sufficient training and knowledge about PGx, and 94% agreeing professional guidelines about PGx use in psychiatry were “unclear or not clear at all.”

#### Incorporation of Pharmacogenetics Into Current Workflow

An issue raised in five of the studies (Dunbar et al., 2012; Shishko et al., 2015; Goodspeed et al., 2019; Vest et al., 2020; Laplace et al., 2021) was uncertainty about how to incorporate PGx into current workflow processes. Vest et al. (2020) found participants felt PGx would be difficult to adopt into current practice, with concerns over finding the time to educate patients about PGx during a consultation. Delaying prescribing, by 3 days in this study, while waiting for PGx results was also expressed as a concern when compared to standard current practice. Moreover, Dunbar et al. (2012) found the average wait for results was 8 days and the upper limit was 42 days. Goodspeed et al. (2019) conducted a focus group with MH clinicians following use of a prototype system reporting PGx data and found users were concerned over how to include PGx reports into current clinical workflow and increased consultation time. How to connect PGx reports to patient medical records was also raised as a point of contention. Similar issues were also raised by clinicians after ordering a PGx test in Dunbar et al. (2012). They also described the delay in receiving the results as being a reason for not using PGx data. Likewise, a small number of psychiatric pharmacists in Shishko et al. (2015) indicated that PGx was too time consuming. Furthermore, 64% of participants in Laplace et al. (2021) were concerned about the delay in prescribing that testing may cause.

#### Misinterpreting Pharmacogenetics Data

Another barrier was the potential for misinterpretation of PGx information. Vest et al. (2020) reported mental health and primary care providers being given PGx results in a traffic-light style report. However, they showed unease over the potential for misinterpretation of this system, especially by patients. Likewise, patients in Liko et al. (2020) demonstrated misinterpretation of PGx reports, the color-coded traffic light system misleading patients that “green bin” medications are drugs that should work and “red bin” to be medications that would not work. Rather these color-coded systems are a guide to the severity of drug-gene interaction.

#### Miscellaneous: Trust, Discrimination and Replacing Clinical Judgment

Subsidiary themes raised less often included concern PGx could replace clinician experience and clinical judgment. Clinicians questioned by Dunbar et al. (2012) expressed reluctance over the idea that PGx test results could take over selection of treatment rather than treating the clinical presentation of a patient. Similarly, a minority of respondents in Laplace et al. (2021) were anxious that PGx could lead to “loss of clinic.”

Clinicians using a prototype clinical decision support tool that incorporated PGx data in Goodspeed et al. (2019), described a lack of trust in the data displayed, stemming from previous experiences using clinical support tools.

There were concerns over the use of PGx data being used to discriminate against patients. Patients surveyed in McCarthy et al. (2020) study of attitudes toward PGx in patients with treatment resistant depression, overall rejected the notion of fearing discrimination based on results. But there were significant differences in race, with non-white groups having overall greater concern about discrimination.

### Enablers

#### Perceived Value of Pharmacogenetics

Most studies found that both HCPs and patients are hopeful that PGx information can improve the precision of prescribing and help guide medication selection. Vest et al. (2020) found primary care and MH providers were hopeful PGx data would help them identify more effective treatments and avoid some of the current trial and error process. Similarly, Chan et al. (2017) found the majority of clinicians surveyed perceived PGx as being useful for identifying suitable medications for treatment. Shishko et al. (2015) found that psychiatric pharmacists believed PGx could be useful in guiding the medication selection and that PGx would become a standard part of clinical practice. Thompson et al. (2015) surveyed psychiatrists and obtained similar findings. Liko et al. (2020) found that prior to testing, the majority of patients were hopeful PGx would help identify the right medication for them. One participant, although skeptical, went on to explain they would expect higher or lower dosage following the use of a psychiatric PGx test. Following the test, some patients perceived it as valuable and another tool to help clinicians choose medication. Likewise, McCarthy et al. (2020) found patients were largely favorable toward PGx testing and strongly endorsed statements about PGx predicting medicines that may be poorly tolerated or less efficacious. Respondents in Laplace et al. (2021) overwhelmingly felt PGx could improve response to treatment with over 98% agreeing on this.

#### Patient Interest in Pharmacogenetics

Patients surveyed in two studies by McCarthy et al. (2020); Kastrinos et al. (2021) had positive outlooks overall on the use of PGx in psychiatry. Kastrinos et al. (2021) demonstrated a high level of interest among patients and positive attitudes toward the use of PGx, while McCarthy et al. (2020) found patients strongly agreed with PGx testing, if it can help them find appropriate treatment and allow patients to plan for the future more effectively.

#### Improving Patient Engagement

Clinicians reported that the use of PGx data can aid the relationship with the patient and make them more open to the use of medication by alleviating concerns relating to the use of medicines. Dunbar et al. (2012) reported that the use of PGx report in consultations helped build trust and rapport between the clinician and patient, acting as a tool for engagement. Similarly, Goodspeed et al. (2019) found PGx reports helped ease patient concerns around starting medication and improved communication of the results. Likewise, Vest et al. (2020) described how clinicians reported PGx testing can increase patient inclination to use medication and overcome resistance relating to medications and side effects.

#### Belief Pharmacogenetics Will Become a Routine Part of Practice

Several of the included studies assessed participants’ beliefs about the future of PGx in psychiatry. Shishko et al. (2015) found only 17% disagreed that PGx would be actively used in clinics/hospitals in 10 years. In Walden et al. (2015), following the use of a PGx test, 80% of practitioners agreed PGx will become standard practice when prescribing psychiatric medications. This is echoed by psychiatrists in Thompson et al. (2015), where 85% believed PGx would become standard treatment. In the same way 70% of participants in Laplace et al. (2021), believed PGx would become routine clinical practice. Some studies go further and attempt to determine what position PGx would have within psychiatric clinical practice. Chan et al. (2017); Goodspeed et al. (2019) found participants would be more likely to use PGx in patients with previous poor tolerability or lack of response in treatment resistant illness. Laplace et al. (2021) echoed this with 66% of participants expressing they would not necessarily use PGx in all depression cases, but 85% would do so in treatment resistant cases.

#### Miscellaneous: Improving Patient Experience With Medications

With respect to the psychological impact of PGx results on patients, two studies found a low risk for negative consequences when receiving the results (McCarthy et al., 2020; Kastrinos et al., 2021). Kastrinos et al. (2021) evaluated psychiatric patient views on PGx, in the context of uncertainty management theory, found patients scored highly for wanting to reduce uncertainty (by receiving results) and low for wanting to escalate uncertainty. While McCarthy et al. (2020) showed patients assessed showed low levels of concern about coping with PGx results, with only 3% endorsing an inability to emotionally cope with the result. This study also showed there was no apparent relationship between severity of depression and ability to cope with PGx results. Doctors surveyed by Laplace et al. (2021) agreed, with fewer than 25% believing there was a risk of psychological distress for patients following PGx testing.

Clinicians were also found to believe use of PGx could help reduce the likelihood of ADRs occurring. Shishko et al. (2015) found over two-thirds of psychiatric pharmacists agreed PGx could reduce the likelihood of adverse events happening. Similarly, clinicians in Dunbar et al. (2012) were found to predict fewer adverse events to be experienced by patients when PGx had informed drug and dose selection. Further comments were made explaining that because of this the therapeutic relationship could improve and adherence would increase.

## Discussion

This review has highlighted a range of barriers and enablers to the uptake of PGx in mental health care settings. Some of these factors are driven by patient beliefs, while others are primarily from a healthcare professionals (HCPs) perspective and some factors are shared by both patients and HCPs.

In line with previous research, cost and funding was identified as a barrier to implementation (McKinnon et al., 2007; Klein et al., 2017). Although this was identified as a barrier, it would be reasonable to expect the cost of pharmacogenetic testing to continue to decrease, making this less of an obstacle in the future. One could also argue cost is a perceived barrier to implementation. A limitation to many of the studies that found cost as a perceived barrier, was that they did not explore cost in depth, such as in the context of cost-effectiveness or cost-benefit. Rather they explored cost in general terms. Cost-effectiveness of psychiatric PGx was beyond the scope of this review, therefore for further information we would refer the reader to a recent systematic review; an economic evaluation on psychiatric PGx (Karamperis et al., 2021). This shows early studies assessing cost-effectiveness are promising but are limited by small sample sizes and high heterogeneity, and as such more research is needed. Therefore, although cost is currently perceived as a barrier to the uptake of PGx in psychiatry, evidence may accumulate in the longer-term supporting the cost-effective use of PGx-informed prescribing, by finding a more appropriate treatment quicker and avoiding the currently deployed trial-and-error approach.

Studies assessing cost savings and cost-effectiveness (Hicks et al., 2015, 2017; Dan Wellings, 2020; Tsermpini et al., 2020; Programme, 2021; van Westrhenen et al., 2021) have been carried out, however are from countries without a publicly funded health service, such as the United States. The implementation of PGx in mental health settings in the United Kingdom would likely be NHS-funded, through existing commissioning pathways. As such, patient perspectives on the cost of using PGx may differ and they may be less concerned about the cost of testing, and potentially concentrate more on whether it is a clinically effective intervention. A 2019 study found 80% of respondents felt the NHS is facing a major or severe funding crisis (Dan Wellings, 2020) and it is possible that the public would support ways to improve efficiency of NHS spending, potentially via improving prescribing in mental health through the cost-effective use of PGx. Whereas in non-taxpayer funded healthcare systems, the patient may be expected to pay for pharmacogenetic testing. Therefore, the debate around the cost of PGx differs slightly in context, dependent on how healthcare is funded in each country.

A lack of knowledge about PGx, particularly in relation to psychiatry, amongst HCPs was identified as another barrier. Opportunities to educate HCPs should be sought, such as the Masters in Genomic Medicine currently funded by the NHS Genomics Education Programme (2021). This will help bridge the gap in knowledge about PGx and help ensure that professionals in the NHS are equipped with the skills to use PGx in practice. Undergraduate courses for HCPs should also look to integrate PGx training. A lack of guidance on the use of PGx was also found. Therefore, guidelines recently published by the Dutch Clinical Psychiatric Association will be a useful starting point for mental HCPs, in addition to those already created by CPIC (Hicks et al., 2015, 2017; van Westrhenen et al., 2021). As more evidence emerges these guidelines should be updated. Studies evaluating the clinical utility of PGx are ongoing, which will add to the evidence-base we currently have on PGx (Tsermpini et al., 2020).

Incorporation of PGx into current practice was also highlighted as a key barrier. HCPs were found to be concerned about how they would find the time to discuss PGx with their patients and the delay in prescribing while they wait for PGx results prior to prescribing medication. A way to facilitate consultations about PGx with patients, would be the development of resources for patients to look at and read. Furthermore, a multi-disciplinary approach, to ensure it is not the sole responsibility of the prescribing clinician to inform the patient about PGx and its associated pros and cons, would help balance the increased workload from PGx across the multidisciplinary team. It is also a possibility that prescribing workload would decrease in the long-term, if PGx can improve the precision and accuracy of prescribing. With the correct training, pharmacists would be well situated to offer patients counseling about PGx and assist prescribers in making PGx-informed prescribing decisions. Future research should aim to evaluate the outcomes of pharmacist led PGx services. Moreover, the model in which the NHS wishes to adopt when implementing PGx in mental health should be explored. Many companies offering PGx testing, also offer clinical decision support (CDS) as part of their service. CDS could overcome the issue of PGx being time consuming for clinicians, by outsourcing the workload. A drawback of this would of course be cost and potential underutilization of clinicians with relevant skills working for the NHS already, such as pharmacists who would be well placed to support prescribers with PGx.

The delay in prescribing differed among the studies undertaking PGx testing. This is possibly due to slower technology being used in older studies, as in the study by Dunbar et al. (2012). But clearly, delaying prescribing while waiting for PGx test results is an issue. If patients are acutely unwell, delaying prescribing may cause more harm than good, if they require prompt treatment. However, an initial slight delay in prescribing, to find the most appropriate medication, could be offset in the long run by reducing the time to therapeutic response by reducing the trial-and-error approach routinely adopted in current practice. Furthermore, the delay in receiving PGx results would likely be overcome following implementation of robust pathways for PGx referrals and large-scale rollout and commissioning of facilities to perform PGx testing for the NHS. Additionally, a medicine could be prescribed with a follow-up consultation planned, to adjust the dose or drug selection based on PGx results once obtained. Pre-emptive testing (van der Wouden et al., 2019) is another model that could be adopted, which would remove delay in prescribing as a barrier to PGx implementation.

Linked to educating patients about PGx, is the potential risk of misinterpreting PGx data which was also identified as a barrier. Again, development of psychiatry specific PGx resources for patients could act as an aid when consulting patients about PGx. Clear explanation of the meaning of PGx reports will be key to overcoming the risk of misinterpretation by patients. Again, pharmacists are well placed to offer this counseling to patients.

A further potential barrier raised by both HCPs and patients was the fear of PGx being used in discriminatory ways. This is a valid concern and future research should ensure that study participants are recruited from more diverse backgrounds, to ensure results of such studies reflect as much as possible the whole population. A limitation to many of the patient studies in this review was that often patients were white and middle-aged (Liko et al., 2020; McCarthy et al., 2020; Kastrinos et al., 2021). Furthermore, a possibility in the future is that new treatments are reserved for those with a particular genotype, in which a drug demonstrates better effectiveness. Exploration of the impact this may have, and patient education will be key to avoid service users feeling discriminated against.

This review found that belief about PGx improving the precision of prescribing resonated amongst patients and HCPs. Current data from trials investigating psychiatry PGx, comparing treatment as usual (TAU) vs PGx-informed prescribing decisions demonstrate improved symptom scores and tolerability (Hall-Flavin et al., 2012, 2013; Winner et al., 2013; Singh, 2015; Pérez et al., 2017; Bradley et al., 2018; Greden et al., 2019). With regards to efficacy, two meta-analyses found PGx-informed treatment for major depressive disorder leads to improved response rate (Rosenblat et al., 2018; Bousman C. A. et al., 2019). However, these meta-analyses were limited by high heterogeneity and risk of bias in the included studies. Therefore, although early studies are promising, data from larger scale studies examining a wider range of MH conditions, using a variety of drug classes and more diverse patient populations are needed to corroborate these early findings. Large scale multi-center trials are ongoing; the hope is that they will provide further insight into the value of PGx in managing MH problems in psychiatry and primary care. More focus on antipsychotics is also required, with current work mainly focusing on assessing the utility of PGx in antidepressant prescribing (Herbert et al., 2018; Tsermpini et al., 2020).

Linked to the finding that clinicians believe PGx can improve the precision of prescribing, is the notion that PGx will become a more routine part of practice. However, a possible limitation to the studies included is the lack of exploration about where PGx may fit into existing care pathways. Although some of the studies made attempts to determine where clinicians see PGx fitting into existing care pathways, an overall perspective was not established. Limited examples from the studies indicate clinicians would be inclined to use PGx in patients with previous poor tolerability or lack of response to medications. Future research should aim to determine practitioner perspectives on this, to help inform implementation of PGx into existing care pathways.

Related to this, is the belief among clinicians that PGx-informed prescribing would lead to fewer ADRs. ADRs contribute 6.5% of hospital admissions in the United Kingdom and ADRs are a leading cause for discontinuing psychotropic medication (Pirmohamed et al., 2004; Velligan et al., 2017). If patients are taking medications that are better matched to their genetic profile, they may experience fewer side effects. A study demonstrated better antidepressant tolerability following PGx-informed treatment selection (Pérez et al., 2017). This study shows that evidence is slowly accumulating regarding PGx improving medication tolerability, which should alleviate concerns relating to a lack of evidence base being a barrier to PGx implementation. Further studies are warranted to investigate if PGx-informed treatment selection improves tolerability of antipsychotics.

Patients were found to have a keen interest in PGx. A key to the successful uptake of PGx in psychiatry in the NHS will be acceptability for patients. The process should include involving patients and the public early on in decisions regarding the implementation of PGx services in the NHS. Further studies should seek to confirm these patient perspectives in users of NHS mental health services.

Regarding the importance of patients being active participants in treatment decisions wherever possible, this review found HCPs believe that PGx could improve engagement and discussions with patients around the topic of medication. Often in psychiatry, patients can be unwilling to take medication, a systematic review found average rate of non-adherence with antipsychotics to be approximately 40% (Lacro et al., 2002). Similar non-adherence rates were found for other psychotropic drug classes (Semahegn et al., 2020). Guiding treatment based on an individual’s genetic profile, could prove invaluable in reaching out to patients hesitant about medication. A study demonstrated that adherence in psychiatric patients improved following a PGx-informed intervention (Fagerness et al., 2014). Further studies should seek to assess the psychological impact on the patient following PGx-informed treatment in the context of their views about medication and their relationship with clinicians.

This review found there was low potential for a negative psychological impact on the patient from having a PGx test. This is plausible, since discovering an individual’s PGx profile predicts only the response to a medication, not a prediction of disease state. Therefore, unlike the potential negative psychological impact of having a genetic test, we would not anticipate such consequences following a PGx test (Oliveri et al., 2018). Patients may be less apprehensive about learning the findings from a PGx test compared with a prognostic genetic test. This is supported by the findings in this review, that patients are generally open to having a PGx test done, more research is needed to validate this.

There are some limitations to the studies evaluated in this review. Firstly, most of the studies in the review were conducted in the United States, which does not have a publicly funded healthcare system. Therefore, the transferability of the findings from these studies needs to be confirmed by conducting studies with HCPs and patients in countries that have publicly funded healthcare systems, such as the NHS in the United Kingdom. Many studies only took on board viewpoints from a limited number of HCPs, such as general practitioners (GPs), psychiatrists, and psychiatric pharmacists. We know that the mental health multidisciplinary team is much larger than these professionals, therefore future research could aim to evaluate the viewpoints of other professions, namely nurses, occupational therapists, social workers, and carers who all play a big role in patient care.

Thus far much of the PGx in psychiatry research has focused on the use of PGx in depression and related to prescribing antidepressants. Further studies should look to broaden the scope of research into PGx in psychiatry, with focus on other drug classes such as antipsychotics and mood stabilizers, in different MH conditions such as schizophrenia and bipolar disorder. Further exploration of antidepressant use in anxiety disorders is also warranted. Investigation of the role of PGx in managing psychiatric conditions in those with physical health conditions and polypharmacy should also be prioritized. Further studies should also focus on evaluating the clinical outcomes of patients who received PGx-informed psychiatric prescribing, for example length of stay in hospital and time spent in illness remission.

Future research should also aim to assess the position of PGx in clinical pathways by exploring which patients would benefit the most from the use of PGx and where in care pathways it is best deployed. It is likely that any uptake of PGx in NHS mental health services will be driven by cost-effectiveness, therefore health economics research will be important in determining the next steps for PGx in psychiatry in the United Kingdom.

Due to this field being specialized, a limitation of the review is that there is only a small number of papers available looking at PGx implementation in psychiatry. Because of this, the study population, country, and clinical setting studied within the included papers are highly heterogenous. As such generalizations made about barriers and enablers identified in this review are limited in nature, due to the similarity in methodologies adopted amongst the included studies. Another limitation is that the review did not compare barriers and enablers in psychiatry to other clinical specialties, this would have been beyond the scope of this review.

## Conclusion

In conclusion, this review provides a detailed analysis of several barriers and enablers to implementing PGx in clinical mental health practice. As the NHS continues to build on the foundation it has laid for the uptake of genomic medicine, services can consider these barriers and enablers when designing care pathways that incorporate PGx. Further research is needed to demonstrate the clinical utility and cost-effectiveness of PGx on a larger scale, and to develop an evidence base for broader range of drug classes. Research in the United Kingdom about patient and HCP perspectives on PGx in psychiatry should seek to validate the data from studies conducted in other countries. Implementation science has a role to play in the integration of PGx into routine mental health clinical practice.

## Author Contributions

AJ contributed to the conception of the mini review, implementing the research, thematic analysis, and writing of the manuscript. SM supervised the process, contributing to the mini review conception, thematic analysis, discussion points, draft revisions and overall oversight, and planning of the mini review. BF and GB contributed to the mini review conception, thematic analysis, discussion points, and draft revisions. AC and GS contributed to mini review conception and draft revisions. CD contributed to draft revisions. All authors contributed to the article and approved the submitted version.

## Author Disclaimer

The views expressed in this article are those of the authors and not necessarily those of the NHS, the NIHR, or the Department of Health and Social Care.

## Conflict of Interest

The authors declare that the research was conducted in the absence of any commercial or financial relationships that could be construed as a potential conflict of interest.

## Publisher’s Note

All claims expressed in this article are solely those of the authors and do not necessarily represent those of their affiliated organizations, or those of the publisher, the editors and the reviewers. Any product that may be evaluated in this article, or claim that may be made by its manufacturer, is not guaranteed or endorsed by the publisher.

## Footnotes

1
https://www.pharmgkb.org/

2
https://cpicpgx.org/

## Supplementary Material

Supplementary Appendix 1 PRISMA searching tool flow diagram.

Supplementary Appendix 2 Table of included studies and their characteristics.
