# Supplementary material for: What Are the Barriers and Enablers to the Implementation of Pharmacogenetic Testing in Mental Health Care Settings?
Source: Front Genet. 2021 Sep 22;12:740216. doi: 10.3389/fgene.2021.740216 (PMC8493030; doi:10.3389/fgene.2021.740216)
Supplement: Supplementary Appendix 1 — PRISMA searching tool flow diagram. [file Table_1.pdf]

Table of included studies and their characteristics					
**Title**	**Author/Year**	**Sample**	**Data Collection**	**Barriers**	**Enablers**
**Primary care and mental health providers' perceptions of implementation of pharmacogenetics testing for depression prescribing**	Vest et al 2020	31 clinicians from primary care and mental health settings.<br><br>(12 x internists; 12 x psychiatrists; 4 x advanced nurse practitioners; 1 x family medicine practitioner; 1 x physician assistant)	Qualitative<br><br>10 x focus groups that explored 10 questions mapped onto different Consolidated Framework for Implementation Research (CFIR) constructs<br><br>Conducted at six sites across the USA participating in the Precision Medicine in Mental Health Care (PRIME) Care study	Lack of knowledge and current evidence base about PGx<br><br>Incorporation into workflow<br><br>Misinterpretation of results	Interest in use of PGx.<br><br>Hope that PGx could lead to precision prescribing.<br><br>Aid clinician relationship with patient
**Patients' Perspectives on Psychiatric Pharmacogenetic Testing**	Liko et al 2020	20 patients, >18 years old, with diagnosis of major depressive disorder or bipolar disorder with depressive symptoms<br><br>95% of participants had either multiple failed pharmacotherapies and/or previous medication intolerance	Qualitative<br><br>Semi-structured interviews – conducted by two researchers asking eight questions covering patient's knowledge of PGx, pre-test expectations of PGx and post-test experience and treatment outcome.<br><br>Conducted at a Depression Center based at the University of Colorado Anschutz Medical Campus	Cost / Funding<br><br>Misinterpretation of results	Interest in use of PGx<br><br>Hope that PGx could lead to precision prescribing.
**PGx in psychiatry: Patients' knowledge, interest, and uncertainty management preferences in the context of pharmacogenomic testing**	Kastrinos et al 2021	598 participants, >18 years old, that self-declared they had taken medication for a mental health (MH) condition.<br><br>Participants were recruited through ResearchMatch, a US national health volunteer registry	Quantitative<br><br>Online survey – containing 45 questions relating to participant preferences for uncertainty management, attitudes towards psychiatric genetic testing, PGx familiarity and interest in participating in PGx testing		Interest in use of PGx<br><br>Low risk of psychological distress from receiving PGx result
**Leveraging the utility of pharmacogenomics in psychiatry through a clinical decision support: a focus study group**	Goodspeed et al 2019	16 x mental health clinicians<br><br>(3 x nurse practitioners; 13 x physicians)	Qualitative<br><br>3 x separate focus groups.<br><br>1st focus group was a guided discussion that aimed to identify desired	Incorporation into workflow<br><br>Trust in PGx results vendor	Aid clinician relationship with patient

		Clinicians were recruited from a Mental Health Center based on Denver, US.	CDS tool aspects, PGx features, determine how clinicians will use PGx data and potential negative consequences of CDS integration.<br><br>2nd focus group was a demonstration of how to use CDS<br><br>3rd focus group was a group discussion about the prototype CDS		
**Clinician's perceptions of pharmacogenomics use in psychiatry**	Chan et al 2017	194 clinicians<br><br>(167 x doctors; 27 x pharmacists)<br><br>Doctors were recruited through their Singapore Medical Council membership, similarly pharmacists through their Institute of Mental Health membership.	Qualitative / Quantitative<br><br>Online questionnaire – containing questions about PGx relating to competency, perceived usefulness, perceived risks, and future education. An open-ended question with free text for comments was also included.	Cost / Funding<br><br>Lack of knowledge and current evidence base about PGx<br><br>Lack of clear guidelines about PGx in psychiatry	Interest in use of PGx.<br><br>Hope that PGx could lead to precision prescribing.
**Psychiatric pharmacists' perception on the use of pharmacogenomic testing in the mental health population**	Shishko et al 2015	91 psychiatric pharmacists from across the USA that were members of the College of Psychiatric and Neurologic Pharmacists	Quantitative<br><br>Cross-sectional survey – included 23 questions mainly opinion based on a Likert scale of 1 to 5	Cost / Funding<br><br>Lack of knowledge about PGx<br><br>Incorporation into workflow	Interest in use of PGx.<br><br>Hope that PGx could lead to precision prescribing.<br><br>Belief PGx will become routine practice.<br><br>Belief PGx could reduce ADRs.
**Attitudes on pharmacogenetic testing in psychiatric patients with treatment-resistant depression**	McCarthy et al 2020	170 veterans (receiving care for depression at San Diego VA or Palo Alto VA between 2015 and 2018) with current depressive symptoms and past treatment failure with at least one previous adequate trial of antidepressant or mood stabilizer	Quantitative<br><br>Questionnaire – covering five dimensions: perceived clinical utility, family's wish to know, planning for the future, coping and fear of discrimination	Fear of discrimination based on PGx results	Interest in use of PGx.<br><br>Hopeful PGx results can guide prescribing decision.<br><br>Low risk of psychological distress from receiving PGx result
**Physicians' opinions following pharmacogenetic testing for**	Walden et al 2015	168 Canadian physicians (either psychiatrists or general	Quantitative		Belief PGx will become routine practice.

**psychotropic medication**		practitioners, who had previously ordered at least one pharmacogenetic test and were taking part in the IMPACT (The Individualized Medicine Pharmacogenomic Assessment and Clinical Treatment) PGx implementation study)	Survey – namely the Pharmacogenetics in Psychiatry Follow-up Questionnaire (PIP-FQ) which collected data about physicians' experience with pharmacogenetic testing, the future of pharmacogenetics and physician demographics		
**Psychiatrist attitudes towards pharmacogenetic testing, direct-to-consumer genetic testing, and integrating genetic counselling into psychiatric patient care**	Thompson et al 2015	113 psychiatrists (members of UCSF Langley Porter Psychiatric Institute)	Quantitative<br><br>Cross Sectional Survey – used five questions to assess attitudes towards integration of genetic counseling into psychiatric care, specifically in the context of the use of pharmacogenetic test results to guide treatment		Hope that PGx could lead to precision prescribing.<br><br>Belief PGx will become routine practice.
**Clinician experiences of employing the AmpliChip® CYP450 test in routine psychiatric care**	Dunbar et al 2012	33 doctors (who had ordered an AmpliChip CYP450 test for a patient during the study period) based within three district health boards serving the greater Auckland region in New Zealand<br><br>(29 x senior medical officers; 5 x registrars)	Qualitative<br><br>Questionnaire – conducted either in person, over the telephone or self-completed by the participant. Questions covered clinicians' experiences of ordering the test and receiving the results, whether they utilized the results, and the perceived advantages and disadvantages of the test	Cost / Funding<br><br>Incorporation into workflow<br><br>Concerns PGx could replace clinical judgement	Hope that PGx could lead to precision prescribing.<br><br>Aid clinician relationship with patient<br><br>Belief PGx could reduce ADRs.
**Acceptability of Pharmacogenetic Testing among French Psychiatrists, a National Survey**	Laplace et al 2021	397 responses from a mixture of psychiatrists and psychiatry residents recruited from mailing lists such as residents' associations, psychiatrist associations, medical boards, hospital centers, local medical associations. Face to face recruitment	Quantitative / Qualitative<br><br>Questionnaire – was developed during several working sessions between research collaborators (at the University of Limoges and Esquirol Hospital Center) and collected sociodemographic and epidemiological data about participants and then focused on questions about PGx acceptance, perceptions, perceived competence, history of training and prescription	Cost / Funding<br><br>Lack of knowledge about PGx<br><br>Concerns PGx could replace clinical judgement.<br><br>Risk of psychological distress for patients<br><br>Fear of discrimination based on PGx results.	Belief PGx could be useful for patients with previously unsuccessful treatment.<br><br>Belief PGx will become routine practice.<br><br>Belief PGx could reduce ADRs.

		was also encouraged.	in PGx, usefulness, intent to use, reliability and ease of use, perceived limits and risks and future training wishes.		
