# Supplementary material for: What Are the Barriers and Enablers to the Implementation of Pharmacogenetic Testing in Mental Health Care Settings?
Source: Front Genet. 2021 Sep 22;12:740216. doi: 10.3389/fgene.2021.740216 (PMC8493030; doi:10.3389/fgene.2021.740216)
Supplement: Supplementary Appendix 2 — Table of included studies and their characteristics. [file Table_2.pdf]

PRISMA 2020 flow diagram for new systematic reviews which included searches of databases and registers only

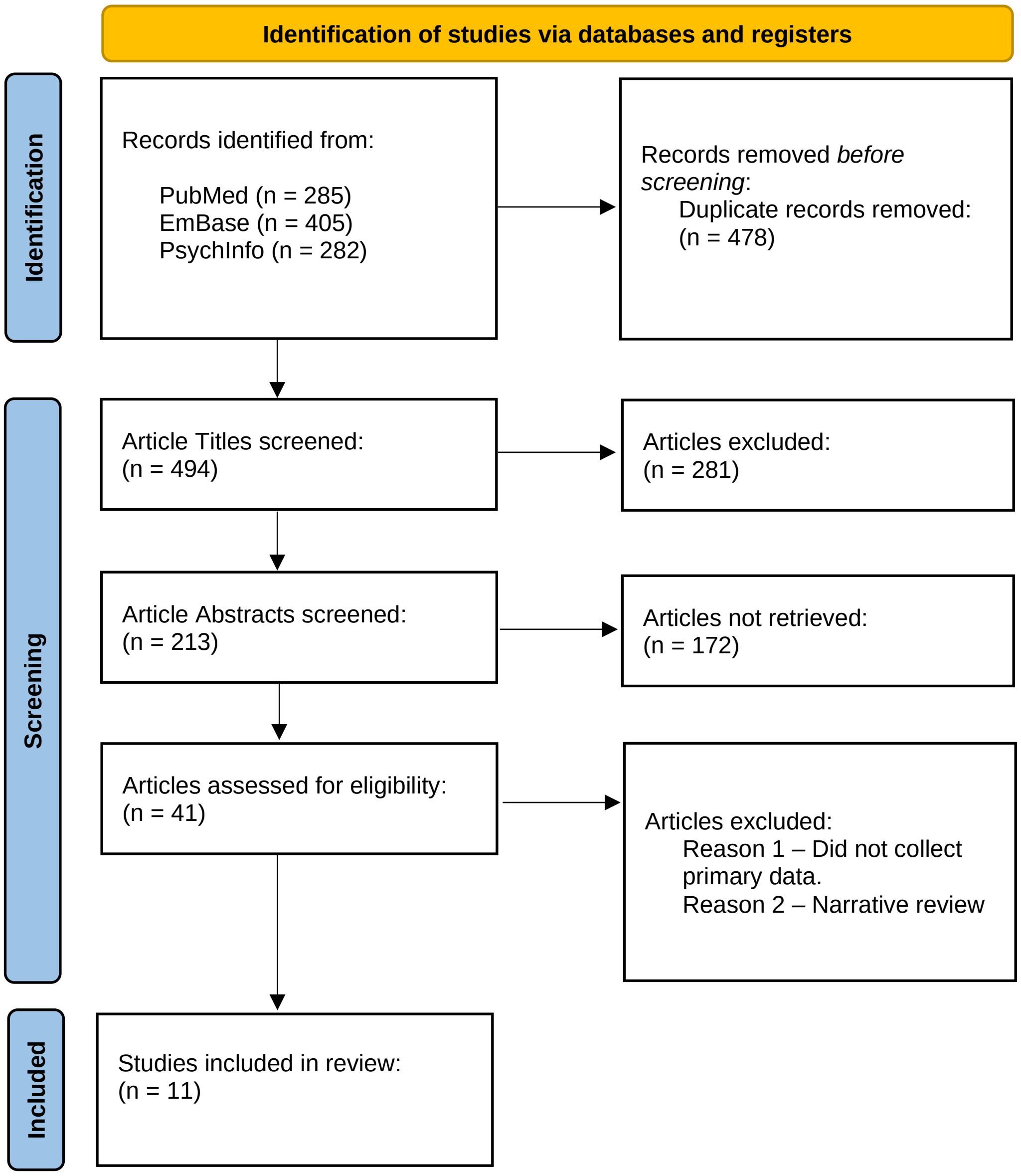

From: Page MJ, McKenzie JE, Bossuyt PM, Boutron I, Hoffmann TC, Mulrow CD, et al. The PRISMA 2020 statement: an updated guideline for reporting systematic reviews. BMJ 2021;372:n71. doi: 10.1136/bmj.n71

For more information, visit: http://www.prisma-statement.org/
